# Prevalence and Factors Influencing Smoking Behavior among Female Inmates in Malaysia

**DOI:** 10.3390/healthcare11020203

**Published:** 2023-01-09

**Authors:** Suharzida Jusoh, Nyi Nyi Naing, Nadiah Wan-Arfah, W.N. Hajidah, Wan Nor Arifin, Ling Shing Wong, Sinouvassane Djearamane, Anto Cordelia Tanislaus Antony, Mohamed Saleem, Siddharthan Selvaraj

**Affiliations:** 1 School of Quantitative Sciences, UUM College of Arts and Sciences, Universiti Utara Malaysia, UUM Sintok, Sintok 06010, Kedah Darul Aman, Malaysia; 2 Faculty of Medicine, University Sultan Zainal Abidin (UniSZA), Medical Campus, Jalan Sultan Mahmud, 20400 Kuala Terengganu, Terengganu, Malaysia; 3 Faculty of Health Sciences, Universiti Sultan Zainal Abidin, Kuala Terengganu 21300, Terengganu, Malaysia; 4 Unit of Biostatistics and Research Methodology, Universiti Sains Malaysia, Kubang Kerian 16150, Kelantan, Malaysia; 5 Faculty of Health and Life Sciences, INTI International University, Nilai 71800, Negeri Sembilan, Malaysia; 6 Department of Biomedical Science, Faculty of Science, Universiti Tunku Abdul Rahman, Kampar 31900, Perak, Malaysia; 7 Department of Chemical Science, Faculty of Science, Universiti Tunku Abdul Rahman, Kampar 31900, Perak, Malaysia; 8 College of Pharmacy, Riyadh ELM University, Riyadh 13244, Saudi Arabia; 9 Faculty of Dentistry, AIMST University, Bedong 08100, Kedah, Malaysia

**Keywords:** smoking, prevalence, factors, prisoners, women inmates

## Abstract

Background: Lately, smoking among adolescents is increasing despite various campaigns to address it being carried out. Previously, this habit was common among men, however, nowadays, smoking has become a habit for women as well. The purpose of this study was to determine the prevalence and its associated factors that influence smoking behavior among women inmates in Kelantan. Methods: A cross-sectional study was carried out among women inmates from Pengkalan Chepa Women’s Prison, Kota Bharu, Kelantan. A total of 274 respondents were needed to answer a self-administered questionnaire. The data were analyzed using Multiple Logistic Regression. Results: A total of 183 participants were smokers. Women who were single and divorced had a lower chance of being influenced to smoke compared to married women. Parents with smoking habits were more associated with children who smoked compared to parents who did not smoke. A participant with secondary level education had higher odds of smoking compared to a participant with primary level education. Smoking peers significantly influenced their friends and, therefore, peer practice was a main factor influencing smoking among women inmates. Conclusion: The prevalence of smoking among women inmates in Kelantan was found to be quite high. Religion (majority (90.5%) of women in the study were Muslims; it would be inappropriate to draw conclusion that religion is an influencing factor), marital status, parents’ practice, peer practice and education significantly influenced women inmates to smoke.

## 1. Introduction

Cigarette smoking is the major single known cause of non-communicable diseases, such as cancer and cardiovascular diseases. Most people try their first cigarette and become daily smokers as adolescents [1]. People who start smoking before 15 years of age have double the risk of developing lung cancer than those who start after the age of 20 years [2]. Smoking is an unhealthy habit, which harms the smokers and also the people close to them when they smoke. Although it is commonly known how cigarettes affect human life, some people ignore the warning. Previously, smoking was a common habit among men, however, nowadays, females, especially adolescents, are taking up smoking too. This habit is getting worse as the number of smokers keeps rising day-to-day. The Global Youth Tobacco Survey by the World Health Organization (WHO) proved that smoking among adolescent females in this country increased from 4.2% in 2003 to 5.3% in 2009, while the number of ex-smokers also increased from 11.5% in 2003 to 12.4% in 2009 [3].

It is estimated that there are 1.15 billion smokers around the world. From that number, more than 80% of smokers are from countries of low and middle income. Approximately 15 billion cigarettes are sold every day or 10 million every minute. It has been estimated that one in five teenagers aged from 13 to 15 years old are smokers. Roughly, 60,000 to 100,000 children in the world start smoking every day and half of them are from Asian countries. In the United States, research has reported that children who are smokers are 13 times more likely to become addicted to drugs compared to children who are not smokers. [3]

‘Health info’ from the Ministry of Health stated that half of Malaysian men are smokers. Every day approximately between 45 and 50 teenagers aged 18 years old start to smoke. In the years from 2005 to 2010, statistics showed an increase in smoking among female adolescents by 8%. Due to this smoking situation, lung cancer cases within this country are increasing by approximately 17% and causing half million coronary cases per year. According to data provided by the National Health and Morbidity Survey—Malaysia [4] and WHO [5], smoking causes approximately 18,000 deaths a year. More than 5 million heavy smokers in this country spend approximately RM 15 million in a day buying cigarettes. This means, that in a year, smokers spend RM 5.8 billion to support their smoking habit.

If this phenomenon continues, there will be many negative consequences in the future. Therefore, this research was conducted to determine the prevalence of smoking behavior among women inmates in Kelantan and to identify its associated factors.

## 2. Materials and Methods

### 2.1. Study Design and Sample

A cross-sectional study was conducted among 274 women inmates at Pengkalan Chepa Prison, Kota Bharu, Kelantan. The subjects included were female prisoners from Pengkalan Chepa Women’s Prison; illegal prisoners were excluded in this study. Due to the limited literature, the sample size of this study was calculated based on the parental smoking factor only. The sample size was calculated using PS Power and Sample Size Software. The parameter used for the calculation was level of significance, *α*, set at 5% and the power, 1-β was set at 80%. Meanwhile, for the probability of the outcome for women who smoked when their parents were not smokers, P_0_ values used were based on the literature review, and for the probability of the outcome in an experimental subject, P_1_ values used were based on expert opinion. The calculated sample size was 264. After adding a 10% non-response rate, the predetermined sample size was 293. Since the available female prisoners were fewer than the predetermined sample size, no sampling method was applied and all women prisoners were included in the study.

### 2.2. Data Collection

In this study, all the information needed was collected using the self-administered questionnaire, which included socio-demographic characteristics, knowledge on smoking hazards, attitude towards smoking tolerance and practice towards smoking. All the respondents were given the consent form before participating in the study and after being informed about the purpose of the study and the confidentiality of data. The researcher distributed the questionnaire to the participants with a brief explanation for self-administration and the response rate was 100%. In this study, a smoker was defined as an individual who had a history of being a smoker or was a current smoker.

### 2.3. Data Analysis

All relevant information on socio-demographic and other associated factors was analyzed using Microsoft Excel 2010 and SPSS version 21. To determine the associated factors influencing smoking among women inmates, SPSS version 21 was used to perform binary logistic regression.

## 3. Results

Overall, 183 (66.8%) of the women inmates were smokers, while the balance, 91 (33.2%), were not smokers. Regarding the reasons for smoking, 25% of smokers said the reason was peer influence, while the reasons for not smoking among 34% of non-smokers was self-discipline. The majority of the respondents (50%) gained information on the hazards of smoking from the media.

The mean (SD = 9.95) age of the respondents was 30.77 years old; the minimum and maximum age was between 16 and 52 years old. The mean (SD = 7.96) age among smokers was 29.85 years old, while the mean (SD = 9.95) age among non-smokers was 32.64 years old. The majority of the respondents were Muslim, which was 248 (90.5%). Regarding marital status, many reported being married (112; 40.9%). The majority of the smokers were married 81 (44.3%), while among non-smokers, the same number of them were divorced as there were who were married (31; 34.1%). A total of 178 (85%) respondents claimed that their parents were married, with most smokers and non-smokers stating that their parents’ marital status was married, which was 65 (71.4%) and 113 (61.7%), respectively. Nearly all the respondents (90.9%) had secondary education (Table 1).

### 3.1. Factors Influencing Smoking among Inmate Women

A total of 146 (53.3%) participants agreed that their parents’ attitude influenced them to smoke. When asked whether their parents’ attitude influenced their smoking habit, 93 (50.8%) of the smokers agreed, while only 53 (58.2%) of the non-smokers agreed. There were 173 (63.1%) respondents who agreed that parental behavior affected them; 132 (72.1%) smokers said they were affected by their parents’ practice, while the majority of the non-smokers (54.9%) disagreed.

From the overall respondents, 154 (56.2%) agreed that body image attitude influenced their smoking, with most smokers (62.8%) reporting that body image attitude influenced them to smoke, but 52 (57.1%) of non-smokers disagreed. Respondents (67.2%) agreed that body image practice contributed to women choosing to smoking. The majority of both smokers (59.6%) and non-smokers (82.4%) said that they were influenced by body image practice. A total of 57.7% respondents claimed that the attitude of peers influenced women to smoke. Smokers agreed that peer attitude had influenced them to smoke (50.3%). For non-smokers, most (72.5%) were affected by their peers’ attitude.

Out of the respondents, 138 (50.4%) agreed that peers’ practice had influenced them to smoke. Among non-smokers, 76 (83.5%) said they were affected by the factor. However, smokers (66.1%) said they were not affected by the factor. Moreover, 160 (58.4%) agreed that the media’s attitude influenced them to smoke. For the smokers, 55.2% of them agreed and 59 (64.8%) of the non-smokers also agreed that the media’s attitude influenced them to smoke. Furthermore, many of the respondents agreed that they were affected by the poor practice of the media (51.8%), with 103 (56.3%) of the smokers agreeing that the poor practice of the media was affecting them to smoke, whereas 52 (57.1%) of the non-smokers said they were not affected by the practice of the media (Table 2).

### 3.2. Multiple Logistic Regression:

Based on the univariate analysis, there were eight significant variables including age, education, parents’ practices, body image practice, body image attitude, peers’ attitude, peer practice and media practice. The multiple logistic regression was analyzed by applying forward selection and backward elimination methods. The variables included in the model after applying the forward selection method were religion, education, parents’ marital status, parents’ practices and peers’ practice. Meanwhile, in backward elimination, there were ten variables left in the model, which were age, religion, education, marital status, parents’ marital status, parents’ practices, parents’ attitude, peers’ practice, body image practice and media attitude. However, based on the parsimonious model concept, the model obtained by the forward elimination method was chosen as the preliminary main effect model. The interactions between variables were not checked because there was no possibility of interactions in the model. No multicollinearity problem existed in the model after the correlation matrix was performed (Table 3 and Table 4).

The goodness of fit of the model was assessed by applying Hosmer–Lemeshow for goodness of fit, the classification table and the area under the ROC curve. It concluded that the model was fit since all of three methods of assessing the goodness of fit of the model showed that the model was fit. The final model was obtained, and the factors were religion, marital status, parents’ practice, peers’ practice and education.

## 4. Discussion

A survey from Global Adults Tobacco Survey [5] by WHO reported that only 1.0% of women from 4.7 million adults currently smoke tobacco, and out of 4.3 million adults, only 0.7% of women currently smoke tobacco daily. From this study, out of 274 women inmates, 66.79% were smokers. As reported by Cropsey et al. [6], they found that 639 out of 866 female prisoners were tobacco users; meanwhile, from 639 respondents, 619 were cigarette smokers [7]. Bryant et al. [8] reported that many studies have found that between 72% and 79% of prisoners are tobacco users.

The factors found to be significant with smoking among women inmates in Kelantan were religion (majority (90.5%) of women in the study were Muslims; it would be inappropriate to draw conclusion that religion is an influencing factor), marital status, peers’ practice, parents’ practice and education. Peer influence is the main factor that influences smoking among women inmates in this study, with many smokers smoking because their friends smoke too [9]. The behavior and choices of peers can influence the behavior and choices of others in their peer group, either a negative or a positive impact, depending on the circumstances. A study conducted by Akers et al. [10] reported that adolescent girls were more influenced in their smoking behavior by their boyfriends than adolescent boys being influenced by their girlfriends [11]. Al Sadat and Bins [12] also reported many women choosing peers as their factor influencing them to smoke because they can make friends through smoking and be accepted in a certain group. Peers and peer relationships have been cited frequently as major factors involved in cigarette use and should be acknowledged in efforts to address the smoking problem [13].

Based on the outcome, secondary level education was associated with higher odds of smoking compared to primary level education. However, Cropsey et al. [6] said education level did not have any significant influence on smoking. Parental smoking also has significance in relation to their children to smoking too [14]. The smoking habits of parents significantly correlates to their children smoking [15]. It has also been proven by Morris [16] that a child aged 11 years old is almost three times more likely to have smoked cigarette in the previous 30 days if they were surrounded by adult smoker. Adolescents are two times more likely to smoke daily if the parents smoke [17]. Another study found that adolescents whose parents are ex-smokers are about one-third less likely to have ever smoked [18]. A few studies conducted have agreed that boys and girls are equally exposed to the effects of smoking parents, which might significantly influence them with regards to smoking [19]. However, some researchers found differences between boys and girls in terms of their receptivity to parental smoking. A study conducted by Aho et al. [20] in 2018 suggested boys were more influenced by parental smoking compared to girls. On the other hand, many studies have said that girls may be more influenced than boys [21,22]. For example, a smoking mother will influence her daughter more towards smoking as daughters tend to follow their mother more compared to the father [23,24].

### 4.1. Significance

This study was an opportunity for the respondents to learn about the hazards of smoking. It could have increased their knowledge and educated them about the consequences of smoking. The study also sought to obtain the main factor that contributes to smoking habits among women inmates to help address the serious smoking problem in society.

### 4.2. Limitations

Although the respondent rate was 100%, there were some respondents who refused to answer the questionnaire because they were illiterate. The study only specifically included respondents who were in Kelantan and did not include individuals from any other states of Malaysia. People who are familiar with smoking will answer based on their experiences, but for those who do not smoke, they will answer randomly.

### 4.3. Recommendation

Authorities and all parties concerned have to work harder to reduce or prevent the public, especially young people, including students, to quit smoking. There are many ways to address the smoking problem, such as campaigns, talking, sports activities and joining a volunteer program [25].

## 5. Conclusions

In conclusion, marital status, peers’ practice, parents’ practice, education, and religion (majority (90.5%) of women in the study were Muslims; it would be inappropriate to draw conclusion that religion is an influencing factor). It is well-known around the world that smoking is dangerous, but people still ignore this as the statistics for smoking keep rising. The My Health Malaysian health ministry web portal statistics show that 23.7 million Malaysians are smokers, and the statistic has increased since it was 3.1 million in 2007 and 4.7 million in 2011. Interestingly, there are many females who are increasingly starting to smoke. They also estimated that every year, 3500 out of every 10,000 deaths in Malaysia are caused by smoking. Therefore, prevention is needed to stop this habit from worsening. Everyone, especially youth, should be involved in interventions as this study showed that peers are the most influencing factor in choosing to smoke.

## Figures and Tables

**Table 1:** Summary of socio-demographic profile among women inmates in Kelantan (n = 274).

Characteristics		Overall	Smoking Status			
			No (n = 91) (33.2%)		Yes (n = 183) (66.8%)	
			Mean (SD)	Frequency (%)	Mean (SD)	Frequency (%)
Age		Mean = 30.77	32.64		29.85	
		SD = 8.75	(9.95)		(7.96)	
Religion						
	Islam	248 (90.5)		80 (87.9)		168 (91.8)
	Buddhism	12 (4.4)		7 (7.7)		5 (2.7)
	Christianity	14 (5.1)		4 (4.4)		10 (5.5)
Marital Status						
	Married	112 (40.9)		31 (34.1)		81 (44.3)
	Divorced	89 (32.5)		31 (34.1)		58 (31.7)
	Single	73 (26.6)		29 (31.9)		44 (24.0)
Parents’ Marital Status						
	Married	178 (65)		65 (71.4)		113 (61.7)
	Divorced	96 (35)		26 (28.6)		70 (38.3)
Education						
	Primary	25 (9.1)		17 (18.7)		8 (4.4)
	Secondary	249 (90.9)		74 (81.3)		175 (95.6)

**Table 2:** Summary of factors influencing smoking among women inmates in Kelantan.

Characteristics	Overall n (%)	Smoking Status, Frequency (%)	
		YES (n = 183)	NO (n = 91)
Parents’ Attitude	Agree = 146 (53.3%)	93 (50.8)	53 (58.2)
	Disagree = 128 (46.7)	90 (49.2)	38 (41.8)
Parents’ Practice	Yes = 173 (63.1)	132 (72.1)	41 (45.1)
	No = 101 (36.9)	51 (27.9)	50 (54.9)
Body Image Attitude	Agree = 154 (56.2)	115 (62.8)	39 (42.9)
	Disagree = 120 (43.8)	68 (37.3)	52 (57.1)
Body Image Practice	Yes = 184 (67.2)	109 (59.6)	75 (82.4)
	No = 90 (42.3	74 (40.4)	16 (17.6)
Peers’ Attitude	Agree = 158 (57.7)	92 (50.3)	66 (72.5)
	Disagree = 116 (42.3)	91 (49.7)	25 (27.5)
Peers’ Practice	Yes = 138 (50.4)	62 (33.9)	76 (83.5)
	No = 136 (49.6)	121 (66.1)	15 (16.5)
Media Attitude	Agree = 160 (58.4)	101 (55.2)	59 (64.8)
	Disagree = 114 (41.6)	82 (44.8)	32 (35.2)
Media Practice	Good = 132 (48.2)	80 (43.7)	52 (57.1)
	Poor = 142 (51.8)	103 (56.3)	39 (42.9)

**Table 3:** Summary of associated factors of smoking among women inmates in Kelantan (Univariate Analysis) (n = 274).

Factor		Smoking Status			B	Crude OR (95% CI)	Wald Test	*p*-Value
		Total	Yes n, (%)	No n, (%)				
Age			29. 85 (7.96)	32.64 (9.94)	−0.036	0.964 (0.937, 0.993)	6.075	0.014
Religion	Islam	248	168 (91.8)	80 (87.9)	0	1		
	Buddhism	12	5 (2.7)	7 (7.7)	−1.078	0.340 (0.105, 10.105)	3.219	0.073
	Christianity	14	10 (5.5)	4 (4.4)	0.174	1.190 (0.362, 3.912)	0.083	0.774
Marital Status	Married	112	81 (44.3)	31 (34.1)	0	1		
	Divorced	89	58 (31.7)	31 (34.1)	−0.334	0.716 (0.392, 1.306)	1.186	0.276
	Single	73	44 (24.0)	29 (31.9)	−0.544	0.581 (0.311, 1.085)	2.902	0.088
Parents’ Status	Married	178	113 (61.7)	65 (71.4)	0	1		
	Divorced	96	70 (38.3)	26 (28.6)	0.437	1.549 (0.899, 2.668)	2.485	0.115
Education	Primary	25	8 (4.4)	8 (4.4)	0	1		
	Secondary	249	175 (95.6)	175 (95.6)	1.614	5.025(2.078, 12.154)	12.837	< 0.001
Parent Attitude	Disagree	128	90 (49.2)	90 (49.2)	0	1		
	Agree	146	93 (50.8)	93 (50.8)	−0.300	0.741 (0.446, 1.231)	1.342	0.247
Parent	No	101	51 (27.9)	50 (54.9)	0	1		
Practice	Yes	173	132 (72.1)	41 (45.1)	1.149	3.156 (1.868, 5.332)	18.459	< 0.001
Body Image	Disagree	120	68 (37.2)	52 (57.1)	0	1		
Attitude	Agree	154	115 (62.8)	39 (42.9)	0.813	2.255 (1.351, 3.763)	9.68	0.002
Body Image	No	90	74 (40.4)	16 (17.6)	0	1		
Practice	Yes	184	109 (59.6)	75 (82.4)	−1.158	0.314 (0.17, 0.581)	13.602	<0.001
Peers’ Attitude	Disagree	116	91 (49.7)	25 (27.5)	0	1		
	Agree	158	9 (50.3)	66 (72.5)	−0.96	0.383 (0.222, 0.660)	11.964	0.001
Peers’ Practice	No	136	121 (66.1)	15 (16.5)	0	1		
	Yes	138	62 (33.9)	76 (83.5)	−2.291	0.101 (0.054, 0.19)	50.377	<0.001
Media Attitude	Disagree	112	82 (44.8)	32 (35.2)	0	1	2.315	0.128
	Agree	158	101 (55.2)	59 (64.8)	−0.403	0.668 (0.397, 1.123))		
Media Practice	No	142	103 (56.3)	39 (42.9)	0	1		
	Yes	132	80 (43.7)	52 (57.1)	−0.540	0.583 (0.351, 0.968)	4.353	0.037

**Table 4:** Associated factors of smoking among women inmates in Kelantan (Multiple Logistic Regression) (n = 274).

Factors		B	Standard Error	Adjusted OR (95% CI)	Wald Test	*p*-Value
Religion	Islam	0		1		
	Buddhism	−3.196	0.836	0.041 (0.008, 0.211)	14.628	<0.001
	Christianity	−0.246	0.813	0.782 (0.159, 3.848)	0.092	0.762
Education	Primary	0		1		<0.001
	Secondary	2.788	0.635	16.249 (4.679, 56.425)	9.268	
Marital Status	Married	0		1		
	Divorced	−1.006	0.403	0.366 (0.166, 0.806)	6.232	<0.001
	Single	−2.045	0.520	0.129 (0.47, 0.359)	15.463	<0.001
Parents’ Practice	No	0	0	1		<0.001
	Yes	1.616	1.616	5.187 (2.511, 10.715)	19.784	
Peers’ Practice	No	0	0	1		<0.001
	Yes	−3.498	−3.498	0.030 (0.011, 0.081)	48.019	

## Data Availability

The corresponding author will provide the dataset of this study upon request.

## References

[1] Nguyen T.T., Hoang M.V. (2018). Non-communicable diseases, food and nutrition in Vietnam from 1975 to 2015: The burden and national response. Asia Pac. J. Clin. Nutr..

[2] Chen Z., Peto R., Zhou M., Iona A., Smith M., Yang L., Guo Y., Chen Y., Bian Z., Lancaster G. (2015). Contrasting male and female trends in tobacco-attributed mortality in China: Evidence from successive nationwide prospective cohort studies. Lancet.

[3] Institute for Public Health (IPH) (2012). Report of the Global Adult Tobacco Survey (GATS) Malaysia, 2011.

[4] Institute for Public Health (IPH) (2012). The National Health and Morbidity Survey: Malaysia Global School-Based Student Health Survey 2012.

[5] World Health Organization (WHO) (2012). Malaysia Releases Its First Global Adult Tobacco Survey. Retrieved Jan..

[6] Cropsey K., Eldridge G., Weaver M., Villalobos G., Stitzer M., Best A. (2008). Smoking Cessation Intervention for Female Prisoners: Addressing an Urgent Public Health Need. Am. J. Public Health.

[7] Jalali F., Hashemi S.F., Babaei A., Abbaspour H., Hasani A., Shakeri H. (2017). The Effectiveness of the Smoking Cessation Programme for Smoker Prisoners Living with HIV/AIDS. Univers. J. Public Health.

[8] Bryant J., Bonevski B., Paul C., O’Brien J., Oakes W. (2010). Delivering smoking cessation support to disadvantaged groups: A qualitative study of the potential of community welfare organizations. Health Educ. Res..

[9] Kozlowski L.T. (2021). Nicotine Addiction, Maurice Seevers, and the First Surgeon General Report on Cigarette Smoking and Health: Conflicting Terms and Interests. J. Stud. Alcohol Drugs.

[10] Akers R.L., Skinner W.F., Krohn M.D., Lauer R.M. (1987). Recent trends in teenage tobacco use: Findings from a five-year longitudinal study. Sociol. Soc. Res..

[11] Kim S., Han S., Maskály J. (2021). Factors of fear of crime among Korean citizens: The mediating effect of confidence in the police. Int. J. Law Crime Justice.

[12] Al-Sadat N., Binns C. (2008). Exploring why girls smoke in Malaysia—A qualitative approach. Asia Pac. J. Public Health.

[13] Henneberger A.K., Mushonga D.R., Preston A.M. (2021). Peer Influence and Adolescent Substance Use: A Systematic Review of Dynamic Social Network Research. Adolesc. Res. Rev..

[14] Montgomery L., Burlew A.K., Korte J.E. (2017). Does change in readiness influence retention among African American women and men in substance abuse treatment?. J. Ethn. Subst. Abus..

[15] Duncan L.R., Pearson E.S., Maddison R. (2018). Smoking prevention in children and adolescents: A systematic review of individualized interventions. Patient Educ. Couns..

[16] Morris G.D., Vo A.N., Bassin S., Savaglio D., Wong N.D. (1993). Prevalence and Sociobehavioral Correlates of Tobacco Use Among Hispanic Children: The Tobacco Resistance Activity Program. J. Sch. Health.

[17] Homel J., Warren D. (2019). The Relationship Between Parent Drinking and Adolescent Drinking: Differences for Mothers and Fathers and Boys and Girls. Subst. Use Misuse.

[18] Holmes C.J. (2018). Today’s decisions, Tomorrow’s outcomes: Does self-control explain the educational smoking gradient?. Soc. Sci. Res..

[19] Rao S., Aslam S.K., Zaheer S., Shafique K. (2014). Anti-smoking initiatives and current smoking among 19,643 adolescents in South Asia: Findings from the Global Youth Tobacco Survey. Harm Reduct. J..

[20] Aho H., Koivisto A.-M., Paavilainen E., Joronen K. (2018). Parental involvement and adolescent smoking in vocational setting in Finland. Health Promot. Int..

[21] Bozzini A.B., Bauer A., Maruyama J., Simões R., Matijasevich A. (2020). Factors associated with risk behaviors in adolescence: A systematic review. Rev. Bras. Psiquiatr..

[22] Pedersen E.R., Miles J.N., Ewing B.A., Shih R.A., Tucker J.S., D’Amico E.J. (2013). A longitudinal examination of alcohol, marijuana, and cigarette perceived norms among middle school adolescents. Drug Alcohol Depend..

[23] Syazmeen R., Latiffah Abd Rani N., Zulkifli A., Latif N., Dobson R., Ibrahim T.A., Semple S., Abidin E.Z., Uny I., O’Donnell R. (2022). Knowledge, Beliefs and Behaviours Related to Second-Hand Smoke and Smoking in the Home: A Qualitative Study with Men in Malaysia. Nicotine Tob. Res..

[24] Wu T.-S., Chaffee B.W. (2020). Parental Awareness of Youth Tobacco Use and the Role of Household Tobacco Rules in Use Prevention. Pediatrics.

[25] Brennan E., Gibson L.A., Kybert-Momjian A., Liu J., Hornik R.C. (2017). Promising Themes for Antismoking Campaigns Targeting Youth and Young Adults. Tob. Regul. Sci..

